# The generation, activation, and polarization of monocyte-derived macrophages in human malignancies

**DOI:** 10.3389/fimmu.2023.1178337

**Published:** 2023-04-18

**Authors:** Paul Chaintreuil, Emeline Kerreneur, Maxence Bourgoin, Coline Savy, Cécile Favreau, Guillaume Robert, Arnaud Jacquel, Patrick Auberger

**Affiliations:** ^1^ Université Côte d’Azur, Institut National de la Santé et de la Recherche Médicale, Nice, France; ^2^ Inserm U1065, Centre Méditerranéen de Médecine Moléculaire (C3M), Nice, France

**Keywords:** monocyte-derived macrophages, CSF-1, differentiation, polarization, TAM, LAM, targeting macrophages

## Abstract

Macrophages are immune cells that originate from embryogenesis or from the differentiation of monocytes. They can adopt numerous phenotypes depending on their origin, tissue distribution and in response to different stimuli and tissue environment. Thus, *in vivo*, macrophages are endowed with a continuum of phenotypes that are rarely strictly pro-inflammatory or anti-inflammatory and exhibit a broad expression profile that sweeps over the whole polarization spectrum. Schematically, three main macrophage subpopulations coexist in human tissues: naïve macrophages also called M0, pro-inflammatory macrophages referred as M1 macrophages, and anti-inflammatory macrophages also known as M2 macrophages. Naïve macrophages display phagocytic functions, recognize pathogenic agents, and rapidly undergo polarization towards pro or anti-inflammatory macrophages to acquire their full panel of functions. Pro-inflammatory macrophages are widely involved in inflammatory response, during which they exert anti-microbial and anti-tumoral functions. By contrast, anti-inflammatory macrophages are implicated in the resolution of inflammation, the phagocytosis of cell debris and tissue reparation following injuries. Macrophages also play important deleterious or beneficial roles in the initiation and progression of different pathophysiological settings including solid and hematopoietic cancers. A better understanding of the molecular mechanisms involved in the generation, activation and polarization of macrophages is a prerequisite for the development of new therapeutic strategies to modulate macrophages functions in pathological situations.

## 1 Introduction

Monocytes are circulating immune cells produced in the bone marrow (BM) during hematopoiesis. This process is responsible for the generation of all blood cells in the bloodstream and ensures their continuous renewal. Hematopoietic stem cells differentiate first into myeloid progenitors that ultimately generate erythrocytes, thrombocytes, granulocytes, and monocytes. Myeloid progenitors give rise to granulomonocytic progenitors and then monoblasts in response to IL-3, GM-CSF (Granulocyte Macrophage Colony Stimulating Factor) or G-CSF (Granulocyte Colony Stimulating Factor). The continuous presence of these cytokines leads to the differentiation of monoblasts into promonocytes and promotes their differentiation into mature monocytes, that leave the BM to enter the bloodstream. The half-life of monocytes in blood circulation is only three days, due to their rapid migration into tissues, their differentiation into macrophages or dendritic cells and their rate of cell death. Detailed analysis of the markers present on the surface of monocytes has revealed different populations of monocytes, mainly characterized by the level of expression of CD14 and CD16 (1, 2). Three distinct monocyte populations coexist in the bloodstream: classical CD14^++^ CD16^-^ monocytes, representing 85% of circulating monocytes, intermediate CD14^+^ CD16^+^ monocytes, accounting for 5% of circulating monocytes, and non-classical CD14^+^ CD16^++^ monocytes, corresponding to the 10% remaining monocytes. Intermediate and non-classical monocytes are derived from classical monocytes and each population is unique in its migration ability, adhesion molecule, cytokine/chemokine, and receptor expression (3, 4). Thus, classical monocytes express CCR2 and CD64, conversely to intermediate and non-classical monocytes, which strongly express CD32 and MHC-II. All these specificities confer these monocyte populations different functions (5–8). Despite these discrepancies, the different monocyte populations share certain functions in common. Monocytes are primarily involved in phagocytosis of cellular debris and circulating pathogens in the blood (9). They all express scavenger receptors that allow them to detect many pathogen-associated molecular patterns (PAMPs) and damaged-associated molecular patterns (DAMPs), including CD14 that recognizes LPS. When a PAMP or DAMP binds to one of these receptors, monocytes become activated and produce various cytokines such as IL-1β, CXCL8 (IL-8) and TNFα as well as reactive oxygen species such as NO (nitric oxide) to eliminate the pathogen or defective self-cells. Monocytes are also capable of presenting antigenic peptides on their surface, although they are much less efficient than specialized antigen-presenting cells such as dendritic cells. Because intermediate and nonclassical monocytes express MHC-II, they can present antigenic peptides more efficiently than classical monocytes and can activate lymphocytes in the bloodstream. One notable difference is that CD16^+^ monocytes express more pro-inflammatory cytokines, such as TNFα, than conventional CD16^-^ monocytes (10). An increase in the CD16+ monocyte population at the expense of CD16^-^ monocytes has also been observed during inflammatory episodes and in pathologies such as atherosclerosis or asthma (11, 12). Conversely, in CMML (Chronic Myelomonocytic Leukemia), classical monocytes accumulate in the blood, show severe activation defects in response to infection or stress and are characterized by alterations in their differentiation into macrophages (13). In addition to their immune functions, monocytes also differentiate into other cell types (macrophages or dendritic cells) in response to different stimuli during their passage through tissues.

Depending on their tissue location, macrophages may endorse a wide heterogeneity of names and functions. Thus, macrophages are called microglia in the brain, osteoclasts and chondroblasts in the bone, Langerhans cells in the skin, Kupffer cells in the liver, alveolar and interstitial macrophages in the lungs (5, 14). However, two types of macrophages are found in the body depending on their origin: resident macrophages derived from embryogenesis such as microglia and macrophages produced from the differentiation of blood monocytes, called monocyte-derived macrophages. Importantly, as the body grows and ages, resident macrophages of embryonic origin are gradually replaced by monocyte-derived macrophages in several tissues, such as in the intestines, kidneys, or the heart (15–17). In addition, resident macrophages can also be replaced, temporarily or permanently, by monocyte-derived macrophages during an inflammatory episode such as a microbial infection (18). This ability of monocyte-derived macrophages to replace and mimic resident macrophages of embryonic origin depends not only on cytokines and growth factors present in the local environment but also on the spatial availability of niches present within the tissue itself. These niches control the number of macrophages present in the tissue and the tissue-specific activity of these macrophages. By regulating the number and functions of resident macrophages, whatever their origins, embryonic and monocyte-derived, these niches play a cardinal function in the regulation of tissue homeostasis of diverse organs (19, 20). Thus, resident macrophages are present in virtually every tissue in the body, where they perform an immune surveillance function and help maintain tissue homeostasis by phagocytosing apoptotic buds and cellular debris. They also perform non-inflammatory functions such as protection of neuronal synapses for microglia or bone destruction by osteoclasts during bone remodeling (21–23). Overall, the role of macrophages, whether of embryonic origin or derived from monocytes, is first to ensure homeostasis in tissues by eliminating dying cells and repairing tissue damage. They also serve to defend the body against pathogens by secreting pro-inflammatory cytokines and by presenting antigenic peptides on their surface.

## 2 The process of differentiation of monocytes into macrophages

### 2.1 From blood circulation to tissues

The differentiation of monocytes into macrophages required signals triggered by cytokines and chemokines secreted by both the injured tissue and the endothelial cells of the blood vessels located in the vicinity of the lesion. The main cytokines and chemokines that allow the recruitment of monocytes are MCP-1 (or CCL2) and MCP-3 (or CCL7), that both recognized the CCR2 receptor expressed on the surface of monocytes. When MCP-1 and/or MCP-3 bind to CCR2, interactions between monocytes and endothelial cells lead to monocyte diapedesis, a process during which monocytes differentiate into macrophages (24). When monocytes are about endothelial cells of blood vessels, they engage interactions through cell surface glycoproteins and selectins expressed on the surface of endothelial cells. Interactions of glycoproteins with E-selectin and P-selectin allow the slowing down of monocytes in the bloodstream. This interaction is strengthened during the process of diapedesis thanks to the expression by monocytes of the integrin receptors LFA-1, Mac-1, or VLA-4. Integrin receptors can strongly bind ICAM1 and VCAM1 integrins, present on the surface of endothelial cells. This interaction eventually leads to the immobilization of the monocyte on the endothelial cells, which consequently remodel their cytoskeleton resulting in the creation of a space between the endothelial cells consecutive to the opening of the cellular junctions. Monocytes thus transit through the blood vessel wall to the tissue via the newly created inter-cellular space (25). It is during diapedesis that monocytes differentiate into macrophages. Indeed, in response to stimuli generated by injured tissue, endothelial cells secrete several cytokines promoting the differentiation of monocytes into macrophages or dendritic cells. Among these cytokines, CSF-1 (M-CSF), CSF-2 (GM-CSF), and IL-34 are all able to induce the differentiation of monocytes into macrophages *ex vivo*, each of them generating macrophages with different functions (26–28). *In vivo*, monocyte differentiation cannot be restricted to the action of these cytokines because the microenvironment around monocytes includes many other cytokines generating monocyte differentiation into different types of macrophages.

### 2.2 Signaling pathways activated downstream of the CSF-1 receptor

**Figure 1:**
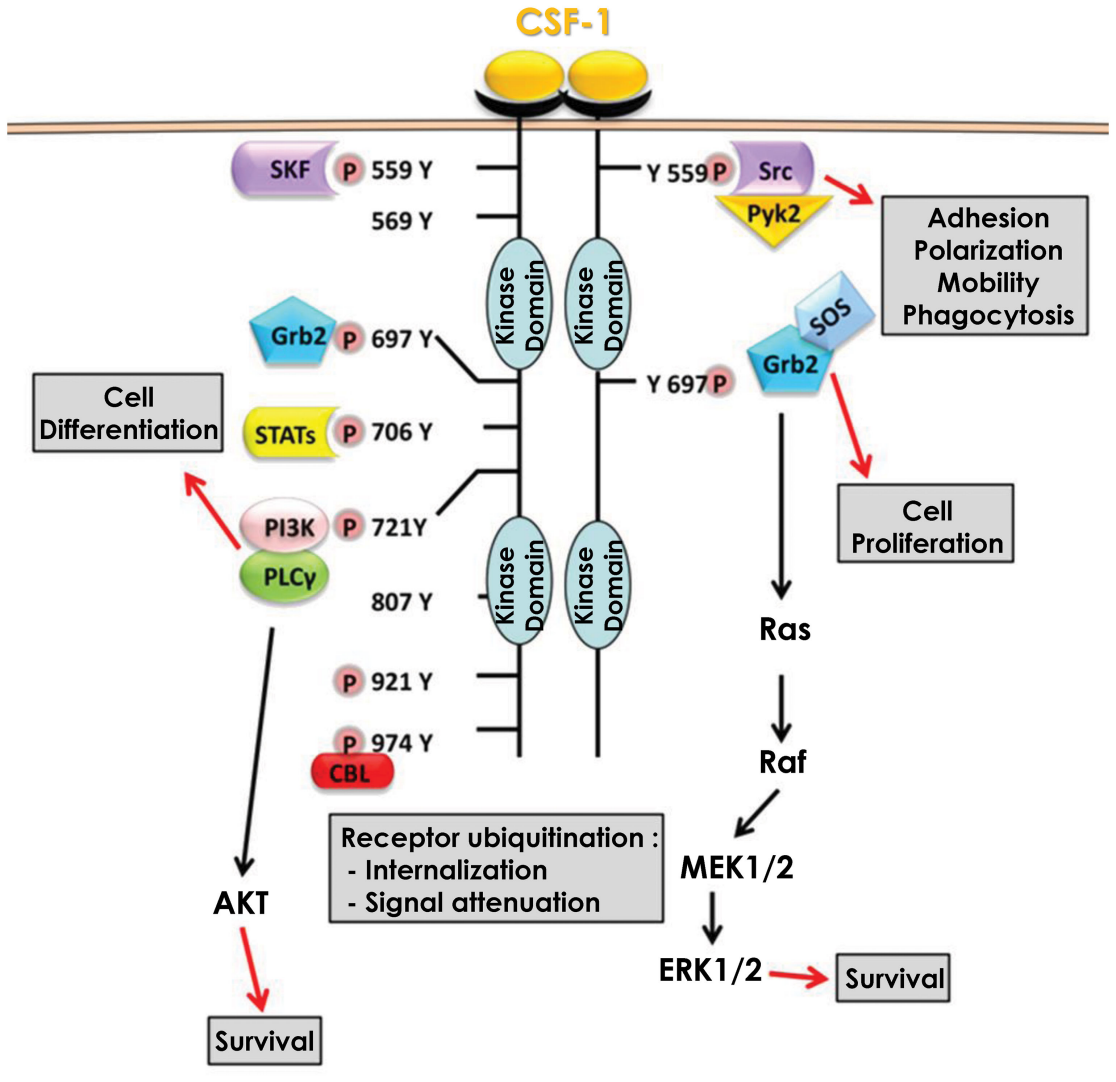
Signaling pathways downstream of CSF1R. Binding of CSF-1 to its receptor CSF1R leads to dimerization and subsequent auto-phosphorylation of several tyrosine residues in the intracellular domain. These phosphorylation reactions are then responsible for the activation of multiple signaling pathways that promote cell differentiation and monocyte survival.

CSF-1 produced by endothelial cells can bind to the CSF1R. CSF1R is a transmembrane receptor with tyrosine kinase activity, expressed weakly in hematopoietic stem cells but much more strongly in monocytes and cells of monocytic origin (macrophages, dendritic cells) (29). It should be noted that the CSF1R can also bind IL-34, another cytokine able to induce the differentiation of monocytes into macrophages (28, 30). CSF-1 binding to its receptor, triggers dimerization and auto-phosphorylation of its intracellular tyrosine residues, resulting in several signaling cascades. Among the activated signaling pathways, some will ensure the survival of the monocyte/macrophage by inducing proliferation and/or inhibiting apoptosis, while others will allow monocytes to differentiate into macrophages (31) (**Figure 1**).

#### 2.2.1 The MAPK/ERK pathway

Activation of the MAPK/ERK pathway occurs via the phosphorylation of tyrosine 697 of the CSF1R (32). This phosphorylation allows the recruitment of two adaptor molecules, Grb2 which recruits RAS and SOS which increases the activity of RAS and then RAF, leading to the activation of the MAPK/ERK pathway (33). This activation which is effective only a few seconds after the binding of CSF-1 to its receptor results in a first phase of transient activation followed by a second phase of continuous activation of ERK (34). However, this second phase is independent of Grb2-SOS and the signaling leading to the activation of the MAPK/ERK pathway under these settings is currently unknown (35). The MAPK/ERK pathway is required for the expression of several proteins including CD33 (Siglec-3), a membrane receptor expressed in cells of myeloid origin that also regulates the expression of Dusp5, a natural inhibitor of the MAPK/ERK pathway that acts as a negative feedback loop of the activation of this pathway (36). CSF-1 also induced expression of VEGF mRNA and protein expression through the MAPK/ERK signaling pathway. Furthermore, CSF-1 triggered nuclear translocation of the transcription factor Sp1 and inhibition of ERK1/2 led to sequestration of Sp1 in the cytoplasm (37). In addition, inhibition of either ERK1/2 or Sp1 leads to a decrease in transcription of the gene encoding VEGF, demonstrating the importance of the MAPK/ERK/Sp1 axis in CSF-1-mediated increase in VEGF expression. Nevertheless, the activation of the MAPK/ERK pathway and its consequences upon CSF-1 binding to its receptor are still largely unknown. Although necessary for monocyte/macrophage survival the roles and cellular targets of the MAPK/ERK pathway during monocyte to macrophage differentiation remains elusive.

#### 2.2.2 The SRC kinase pathway

Activation of the SRC kinase pathway occurs via phosphorylation of tyrosine 559 of the CSF1R (38). This phosphorylation allows the recruitment and direct activation of SRC kinases, that next phosphorylate and activate the kinase PYK2 (39). Following phosphorylation by SRC kinases, Pyk2 is relocated to focal adhesion complexes to phosphorylate paxillin (40). SRC kinases also regulate integrins, particularly the α5 and β1 subunits. Inhibition of SRC kinases results in a significant loss of macrophage motility through their inability to form filipodia, which are required for movement. This loss of movement capacity is partly related to the inactivation of paxillin, which no longer form focal adhesion complexes (41). SRC kinases also activate phospholipase Cγ2 (PLCγ2) and the MAPK pathway in a delayed manner. Indeed, both PLCγ2 and MAPKs exhibit a first transient activation phase quickly after CSF1R activation and then a second persistent activation phase several hours later. Inhibition of SRC kinases at the onset of CSF1R activation leads to a loss of PLCγ2 and MAPK phosphorylation only during the persistent activation phase but does not affect the phosphorylation that occurs during the transient phase. SRC kinase inhibition next leads to an abrogation of the differentiation of monocytes into macrophages (42). Furthermore, Lyn and Hck, two members of the SRC kinase family, are involved in caspase activation during differentiation, their inhibition leading to a decrease in Nucleophosmin (NPM) substrate protein cleavage (please refer to the caspase activation cascade below). However, the mechanism of action of Lyn and Hck during caspase activation is not yet determined, although it is hypothesized that Hck regulates the activation of the PI3K/AKT waves (43).

#### 2.2.3 The PI3K/AKT pathway

Activation of the PI3K/AKT pathway occurs via the phosphorylation of tyrosine 721 of the CSF1R (44). This phosphorylation allows the recruitment and direct activation of PI3K, which then associates with and activates PLCγ2 (45). At the same time, PI3K triggers AKT activation and downstream signaling pathways (46). Inhibition of the PI3K/AKT pathway leads to death of differentiating monocytes by apoptosis, suggesting that the PI3K/AKT pathway is responsible for cell survival during macrophage differentiation. This cell death pathway, which is also dependent on caspases-8, -3 and -9, is blocked during differentiation by the increased expression of anti-apoptotic proteins such as Bcl-xL and Mcl-1 (47). Increased expression of Bcl-xl and Mcl-1 depends on PI3K/AKT activity as its inhibition reduces Bcl-xL and Mcl-1 levels and increases expression of the pro-apoptotic protein Bax. Similarly, inhibition of NF-κB leads to a reduction in Bcl-xL expression and death by apoptosis of differentiating monocytes. The induction of NF-κB, and in turn of Bcl-xL during differentiation is dependent on the ability of PI3K/AKT to degrade IκB, the natural inhibitor of NF-κB. Indeed, when PI3K/AKT is inhibited, IκB is no longer degraded and thus can repress NF-κB, leading to cell apoptosis. Thus, the PI3K/AKT/NF-κB axis ensures cell differentiation by protecting monocytes from apoptosis partly through Bcl-xL (48). AKT activation occurs in successive waves and is concomitant with the phosphorylation/dephosphorylation process of the CSF1R tyrosine 721. These waves of activation, which increase in duration and strength with each wave, are necessary for the differentiation of monocytes into macrophages and promote the activation of non-apoptotic caspases, another major pathway of macrophage differentiation (43).

#### 2.2.4 The caspase cascade pathway

**Figure 2:**
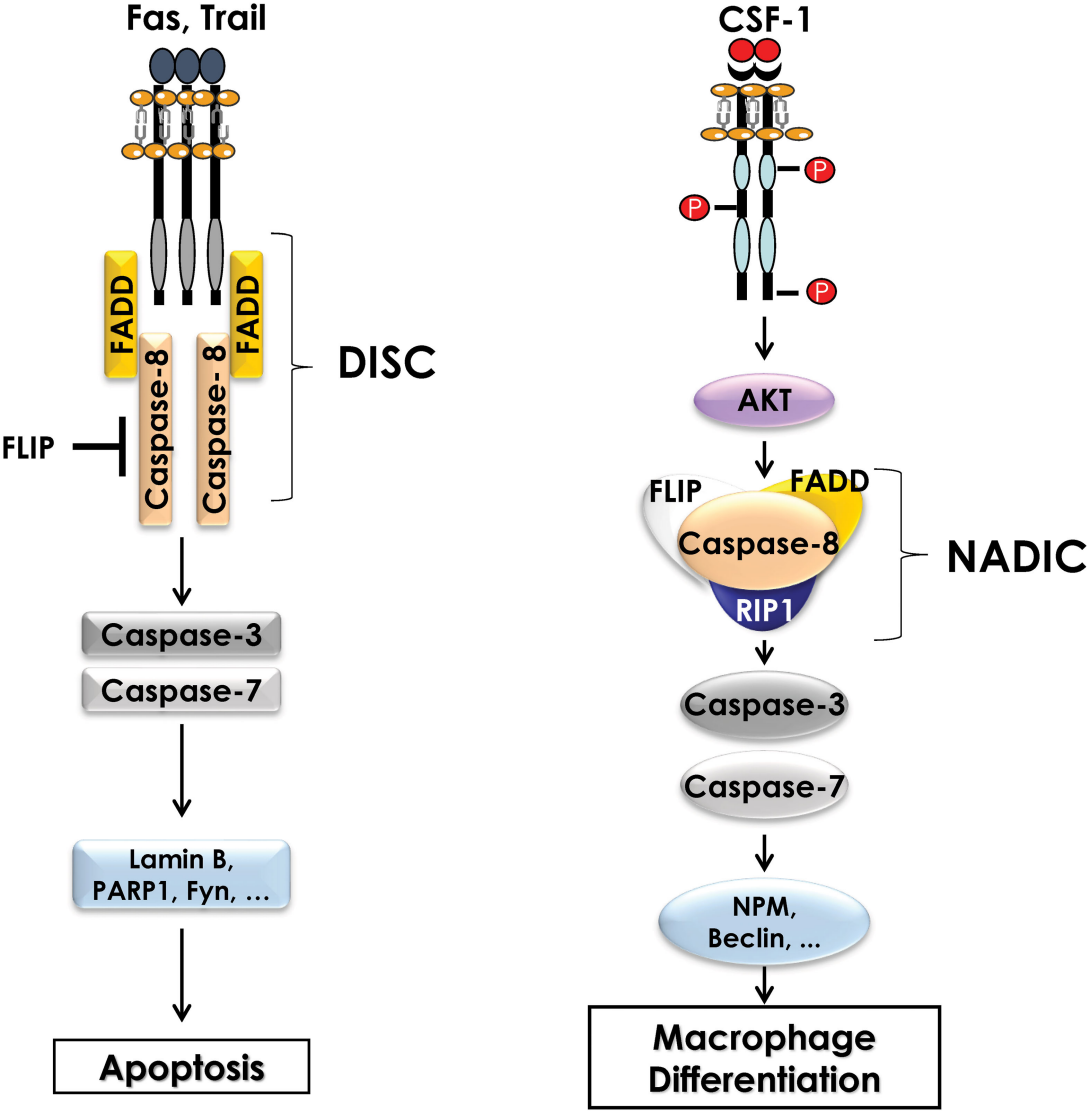
Comparison of the Death Inducing Signaling Complex (DISC) and the complex formed during differentiation of monocytes (NADIC). During induction of extrinsic apoptosis, caspase-8 interacts with a death receptor (FAS, TRAIL) and FADD to form the DISC (Death-inducing signaling complex) at the cell membrane. The FLIP protein may also be present in the DISC to inhibit caspase-8 activity. Activation of caspase-8 leads to the cleavage of caspases-3 and -7 and then to the cleavage of numerous substrate proteins, ultimately inducing apoptosis of the cell. During the differentiation of monocytes into macrophages, CSF1R triggering leads to the activation of the AKT pathway, which is involved in the formation of an intracellular multimolecular complex including caspase-8, FLIP, FADD and RIP1 called non-apoptotic differentiation-inducing complex (NADIC). Within this complex, caspase-8 is activated and then cleaves caspases-3 and -7 to allow the cleavage of substrate proteins that are required for differentiation of monocytes into macrophages.

The activation of non-apoptotic caspases during the differentiation of monocytes into macrophages was first demonstrated in primary human monocytes. Indeed, in response to CSF-1, caspases-3 and -9 are activated without inducing apoptosis. Using the human cell line U937 as a model, and phorbol esters as a differentiating agent, it was reported that caspase inhibition impaired monocyte differentiation and triggered caspase-independent cell death (49). Later, caspase-8 was shown to be important for differentiation using caspase-8 deficient mice. Indeed, *ex vivo* stimulation of myeloid progenitors from caspase 8 knock-out mice with CSF-1 resulted in a significant decrease in the number of differentiated macrophages compared to the one of wild-type mice (50). These results highlighted the important role played by caspases during the differentiation of monocytes into macrophages, but their mechanisms of activation and action has remained unknown for a long time. Only recently, data from our group underscored an original mode of caspase activation during the differentiation of monocytes into macrophages. Indeed, in response to CSF-1, caspase-8 is activated, generating a 34 kDa cleavage fragment, different from the fragments detected following induction of apoptosis suggesting different functions for caspase-8 during differentiation and apoptosis. During monocyte differentiation, activation of caspase-8 occurs within a multimolecular complex, we called Non-Apoptotic Differentiation Inducing Complex (NADIC) composed of the long isoform of FLIP (FLIP-L), FADD and RIP1. This complex is reminiscent of the DISC that assembles during apoptosis but unlike the later does not include a death receptor (**Figure 2**). In NADIC complex, caspase-8 cleaves FLIP and RIP1, generating cleavage fragments of 43 kDa (found in other contexts) and 42 kDa (only found during the differentiation of monocytes into macrophages) respectively, giving them new functions. The cleavage fragment of FLIP allows a more efficient recruitment of RIP1 into the complex while the cleavage fragment of RIP1 negatively regulates the activation of NF-κB, which is transient during the differentiation of monocytes into macrophages. Caspase-8 activation leads to the cleavage of caspase-3 in an original 26 kDa fragment that is absent during the completion of apoptosis. The importance of caspase-8 in the cleavage of caspase-3 has been demonstrated using siRNAs directed against this protease (43, 51). Next, to identify the substrates of caspases during the differentiation of monocytes into macrophages, U937 cells were stably transfected with an empty vector or with a vector containing the p35 protein a natural and irreversible inhibitor of caspases. Following phorbol ester stimulation of U937 cells to induce differentiation, 2D-gel electrophoresis and mass spectrometry analyses were performed. By comparing the proteomes of control and phorbol ester-treated cells, dozen proteins were identified as potential targets of caspases. Among these proteins, some were cytoplasmic, such as RIP1, while others were nuclear, such as (NPM). Most of these proteins are cleaved by caspase-3 but some of them are cleaved by caspase-8 (RIP1, FLIP) (52).

Although the function of the cleavage fragments of RIP1, FLIP and NPM during the differentiation of monocytes into macrophages has been investigated, the role of other caspase substrate proteins is still under investigation. The function of RIP1 and FLIP having been discussed previously, we will only mention here the role of the NPM protein. NPM is a protein expressed ubiquitously in the body and has several functions, including centrosome duplication and ribosome regulation. NPM is also a chaperone protein belonging to the NF-κB complex where it plays a co-activator or co-repressor role depending on the transcriptional targets. During differentiation, NPM is cleaved by caspases, generating a 30 kDa cleavage fragment and then by cathepsins to yield a 20 kDa cleavage fragment. Our recent data established that the cleavage site of NPM by caspases during differentiation of monocytes occurs in a specific sequence, different from the apoptotic consensus sites of apoptotic caspases (Chaintreuil et al., unpublished observation). NPM cleavage is not necessary for the differentiation of monocytes into macrophages but is required for the functionality of the generated macrophages. Indeed, overexpression of NPM cleavage fragments results in a reduction of the phagocytic capacity and motility of macrophages (53). P47^PHOX^ is another substrate of caspases during differentiation of human macrophages. Our recent results established that P47^PHOX^ cleavage by caspase-7 promotes the formation of the NAPDH complex NOX2 and the production of cytosolic superoxide anions (54).

#### 2.2.5 The autophagy pathway

**Figure 3:**
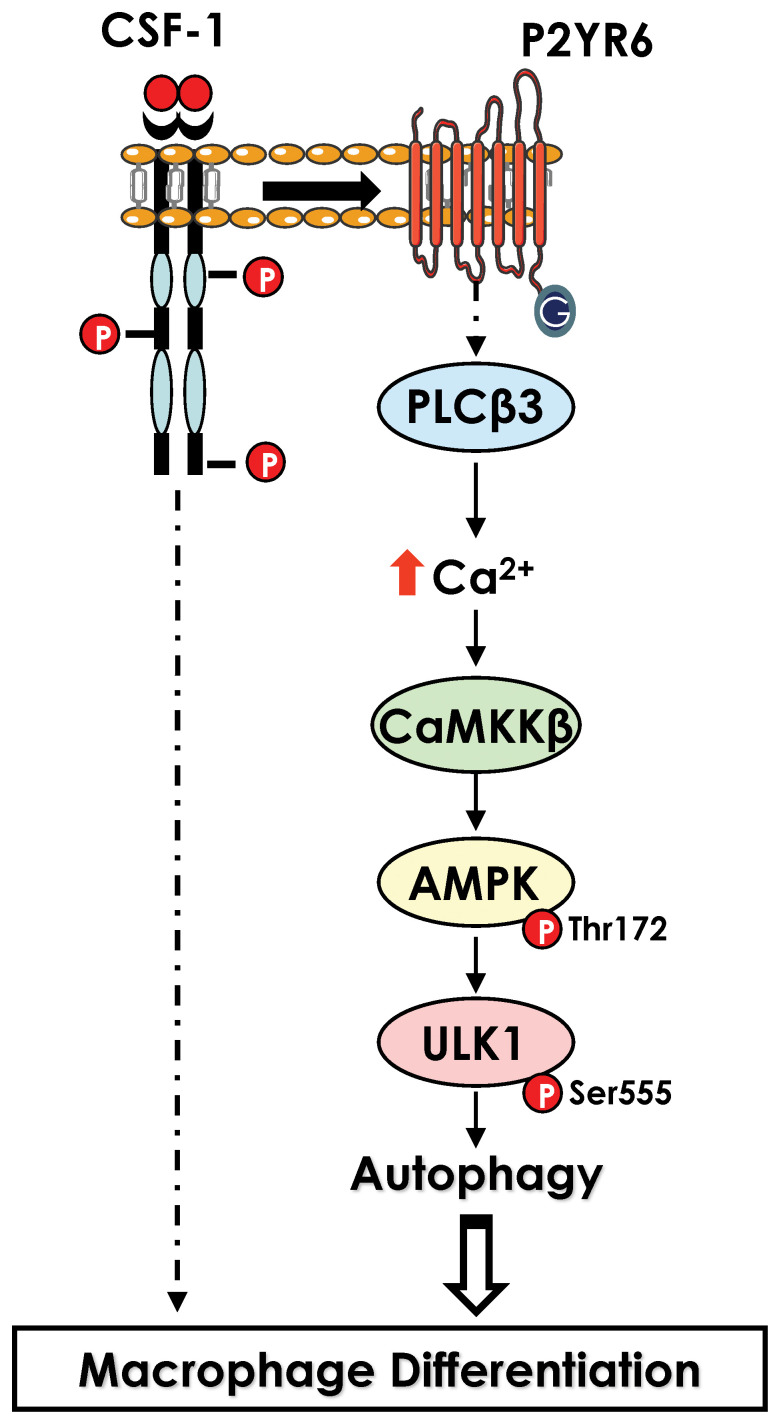
Activation of autophagy during macrophage differentiation. Activation of CSF1R leads to an increase in level of the purinergic receptor P2RY6 at the surface of differentiating monocytes. P2RY6 triggers PLCβ activation, increase in calcium level and a cascade of kinase activation CAMKK2>ULK1 that culminate in the induction of autophagy.

In monocytes stimulated with CSF-1, electron microscopy analysis has revealed a drastic increased in the number of phagosomes and auto-phagolysosomes and characteristic images of mitophagy in differentiating monocytes. Accordingly, we found that LC3-I was lipidated, and cleaved to LC3-II, a hallmark of autophagy induction, while cathepsin B activity and LAMP2 protein expression were drastically increased. In addition, ULK1, a kinase involved in the initiation phase of autophagy, was phosphorylated by CAMKK2, leading to an increase in its activity. Accordingly, pharmacological, or genetic inhibition of CAMKK2 and ULK1 resulted in a defect of monocyte differentiation into macrophages. Moreover, pharmacological inhibition of cathepsins or genetic inhibition of different ATG genes including Beclin-1 (ATG6) or ATG7 also impaired macrophage differentiation, demonstrating the essential role of autophagy in this process (55). Interestingly, in CMML, monocytes exhibit significant differentiation defects, due in part to the presence of α-defensins in the blood of patients. This observation led to the identification of the receptor responsible for autophagy induction in CSF-1-stimulated monocytes. Indeed, the purinergic receptor P2RY6 is inhibited by α-defensins, leading to a defect in the differentiation of monocytes into macrophages. We established that CSF-1 increased P2RY6 expression and activity at the cell surface, leading to activation of a PLCβ3 - CAMKK2β - AMPK - ULK1 signaling pathway responsible for the induction of autophagy. Accordingly, genetic inhibition of each of these proteins individually results in an impairment of autophagy induction and macrophage differentiation (55, 56)(**Figure 3**).

## 3 The process of macrophage polarization

**Figure 4:**
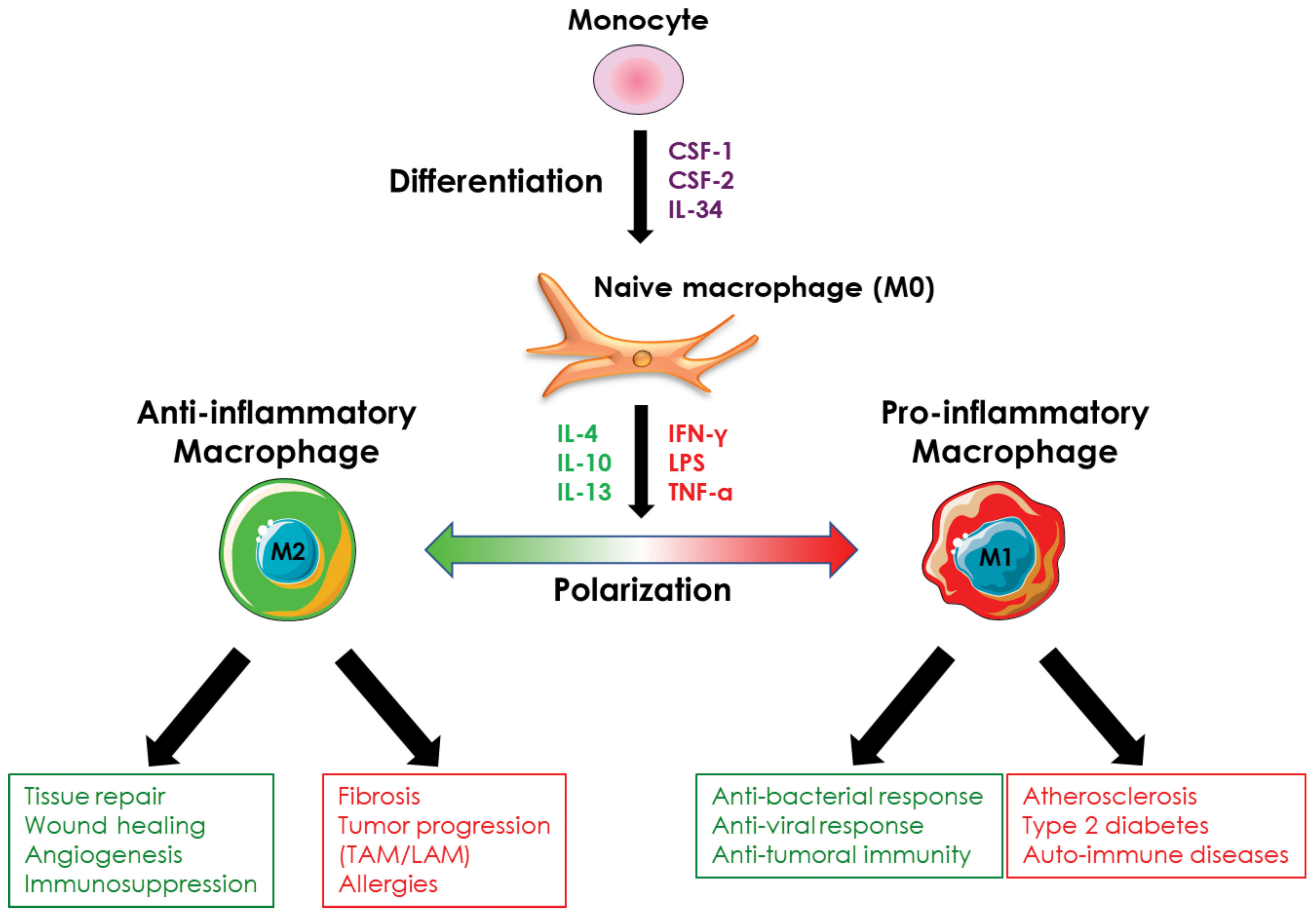
Macrophage polarization. Monocyte-derived-macrophages can be polarized *ex vivo* and *in vivo* into pro- or anti-inflammatory macrophages in response to different stimuli. Pro-inflammatory macrophages are involved in the anti-microbial and anti-tumor response and are implicated in the evolution of several pathologies such as atherosclerosis or type 2 diabetes. Anti-inflammatory macrophages exhibit an immunosuppressive activity, are involved in tissue repair, and promote angiogenesis. They are also involved in the evolution of various pathologies including fibrosis and tumor progression.

*Ex vivo*, macrophages stimulated with CSF-1 are considered naïve (also called M0 macrophages) and, although they possess phagocytic activity and pathogen detection ability, they must undergo a polarization process to acquire their full functions, whether pro- or anti-inflammatory. *In vivo*, the recruited monocytes are differentiated and polarized simultaneously according to the extracellular environment and the secretion of cytokines by the different cell types present at the site of monocyte recruitment. This cytokine environment controls the functions of macrophages, whatever their origin, so that they can respond spatially and temporally to the tissue conditions they are confronted with. Thus, *in vivo*, macrophages encompassed a broad diversity of cells with different roles (anti-inflammatory or pro-inflammatory) and functional states that are determined by microenvironmental signals (14, 57, 58). This balanced phenotype applies to both resident and monocyte-derived macrophages. However, *ex vivo*, it is difficult to mimic the complex extracellular environment that allows monocyte-derived macrophages to exhibit a large spectrum of profiles between pro- and anti-inflammatory and a plethora of different functions. Thus, to specifically study monocyte/macrophage differentiation or polarization, it is possible *ex vivo* to dissociate monocyte differentiation from macrophage polarization by sequentially adding the cytokine(s) that promote differentiation of monocytes into macrophages and the ones that trigger specialization/polarization of macrophages. Thus, the polarization of macrophages *ex vivo* allows the generation of macrophages with a pro-inflammatory profile (M1 macrophages) or, conversely, with an anti-inflammatory profile (M2 macrophages) depending on the cytokines used (**Figure 4**). Although the dichotomy between pro- and anti-inflammatory macrophages is increasingly being challenged *in vivo*, *ex vivo* experiments that use simplified models of pro- or anti-inflammatory macrophage polarization, remain pertinent at least to decipher the molecular mechanisms involved in macrophage polarization. The following sub-sections, therefore, encompass the knowledge acquired on macrophage polarization *ex vivo* and *in vivo*.

### 3.1 Pro-inflammatory polarization

Several stimuli allow naïve macrophages to display a pro-inflammatory climate, among which the co-stimulation with LPS and IFNγ or TNFα. *In vivo*, IFNγ and TNFα are mostly secreted by Th1 T cells that condition a pro-inflammatory environment. Pro-inflammatory macrophages also express and secrete numerous pro-inflammatory cytokines including TNFα, IL-1β, IL-6, IL-12, GM-CSF, which amplify inflammation. Secretion of CCL20, CXCL10 and CXCL11 by pro-inflammatory macrophages increases the recruitment of certain immune cells including Th1 T cells, neutrophils, and NK cells (59). Pro-inflammatory macrophages also express on their surface specific markers such as MHC-II or CD80 and CD86, which are co-stimulatory proteins that participate in T cell activation. Pro-inflammatory macrophages have mainly bactericidal, virucidal and anti-tumor activity. In addition to their ability to secrete various cytokines and chemokines, they also generate ROS via activation of the NADPH oxidase complex and phagocytose pathogenic organisms via the complement cascade. Phagocytosis removes bacteria and viruses from the extracellular environment, while ROS generation leads to tissue degradation. This is an extremely efficient process that allows the elimination of infectious elements (bacteria, viruses) or cancerous cells present at the expense of tissue integrity. To prevent too much tissue damage, the pro-inflammatory response is inhibited by specific immune cells, including Th2 T cells and anti-inflammatory macrophages (60).

### 3.2 Anti-inflammatory polarization

**Figure 5:**
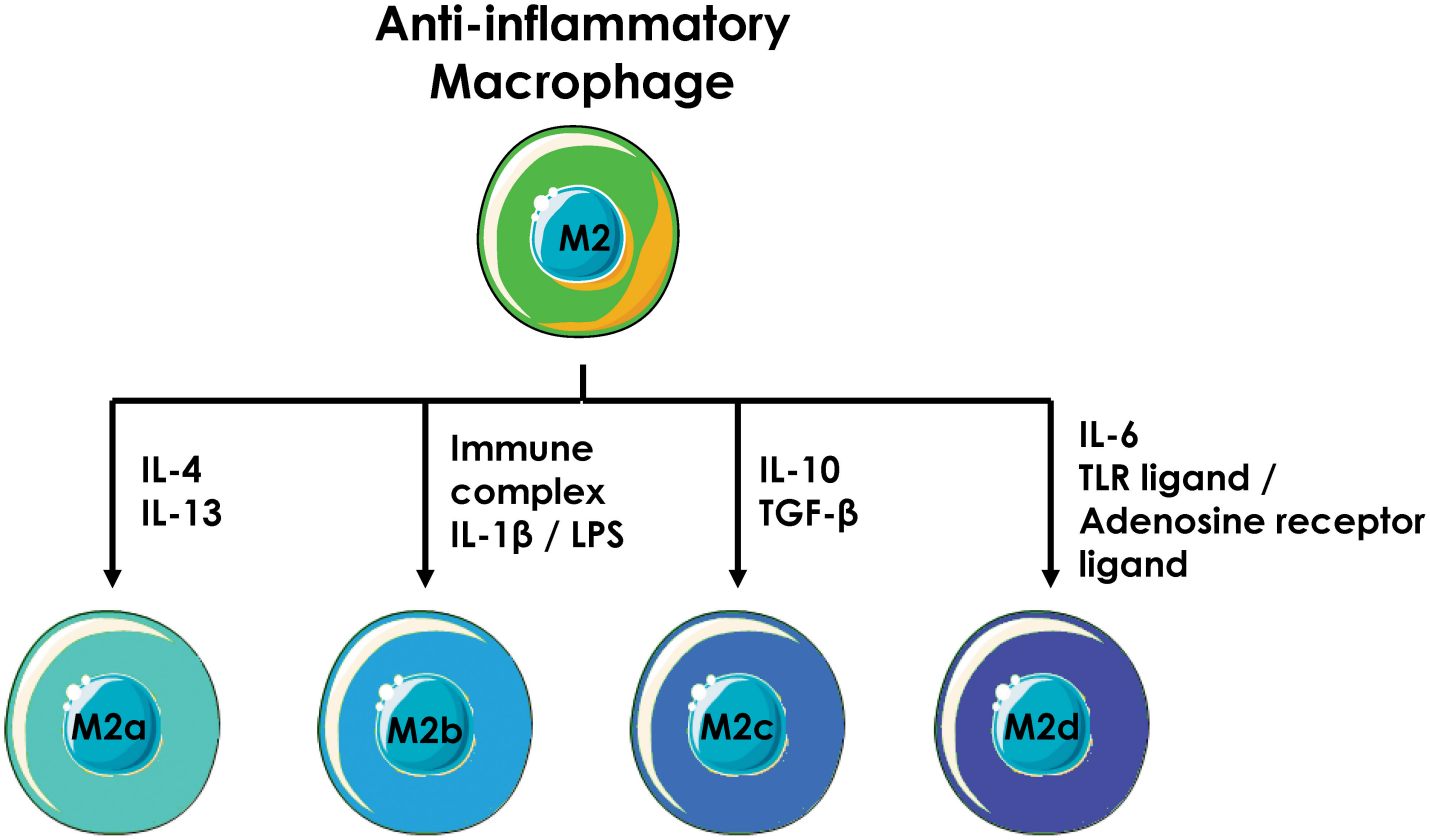
Anti-inflammatory macrophage polarization. Anti-inflammatory macrophages are divided into four subpopulations, each of which is generated by different stimuli.

Anti-inflammatory macrophages are subdivided into four main subtypes: M2a, M2b, M2c, and M2d. Each expresses specific markers, produces distinct cytokines, and exerts different functions. Their polarization status is heavily dependent on different cytokines secreted by Th2 T cells (**Figure 5**).

#### 3.2.1 M2a macrophages

M2a macrophages are generated following the stimulation of naive macrophages with IL-4 and/or IL-13. These macrophages express on their surface receptors such as CD163, CD206 or CD209 which bind cellular debris, giving them a strong phagocytic potential. They express and secrete anti-inflammatory cytokines and chemokines such as IL-10, TGF-β, CCL17, CCL24 and CSF-1 which limit inflammation by inhibiting pro-inflammatory immune cells and recruiting more anti-inflammatory cells such as Th2 and Treg (regulatory T lymphocytes) (61). During the generation of M2a anti-inflammatory macrophages, IL-33 potentiates IL-13-induced polarization by increasing the expression of arginase-1, CCL17 and CCL24 (62). M2a macrophages have mainly immunosuppressive activity and are involved in tissue repair. Their phagocytic potential allows them to remove cellular debris following inflammation, thus favoring injured tissue repair. Arginase-1 plays an important role in this process as it catalyzes the modification of L-arginine to L-ornithine, which then yields polyamines and proline, two compounds necessary for tissue healing and repair (63). They also secrete proteases (mainly MMPs) that degrade necrotic tissue before replacement with healthy tissue (64).

#### 3.2.2 M2b macrophages

M2b macrophages are derived from naive macrophages co-stimulated with an immune complex (a complex formed by an immunogenic epitope and an antibody) and an agonist of TLRs (LPS) or IL-1 receptor (IL-1β) (65). M2b macrophages exhibits a balanced profile between pro- and anti-inflammatory and express various pro-inflammatory (TNFα, IL-6, IL-1β…) and anti-inflammatory (IL-10, CCL1, CCL17….) cytokines. They express CD86 and participate in T cell activation (66). Despite a strong phagocytic activity, M2b macrophages are not involved in bacterial clearance, this role going to M1 macrophages. By contrast, they can inhibit the polarization of naive macrophages into pro-inflammatory macrophages, thus promoting the survival of bacteria, viruses, or cancer cells (67). CCL1 expression by M2b macrophages is essential because it not only maintains M2b macrophage functions but also preferentially recruits Th2 and Treg T cells over Th1 T cells to mediate an anti-inflammatory response (68, 69).

#### 3.2.3 M2c macrophages

When naive macrophages are stimulated with IL-10, they polarize into M2c macrophages and express extracellular receptors such as CD206, CD163 or MerTK (Mer Tyrosine Kinase) on their surface. They secrete anti-inflammatory cytokines and chemokines (IL-10, CCL18, CXCL13, TGFβ, etc.) that help limit the development of inflammation (70). M2c macrophages are primarily involved in tissue homeostasis. They have a strong anti-inflammatory activity mediated by cytokines and chemokines as well as the ability to phagocytose apoptotic cells via the MerTK receptor. This receptor can bind two ligands: Gas6 and Protein S. Gas6 and Protein S bind to negatively charged phospholipids such as phosphatidylserine which is exposed at the level of the outer leaflet of the plasma membrane of the cell during apoptosis. Thus, when the cell enters apoptosis, Gas6 and protein S are exposed outside of the cell and bind to MerTK, leading to phagocytosis of the apoptotic cell by the M2c macrophage (71, 72). This phagocytic ability is important to maintain cellular homeostasis by rapidly removing apoptotic buds (73).

#### 3.2.4 M2d macrophages

M2d macrophages have been identified recently and correspond to the population of immunosuppressive macrophages found in cancers. They are more generally known as TAM (Tumor-Associated Macrophages) or LAM (Leukemia-Associated Macrophages) (74–76). However, this designation is not perfectly accurate as TAMs and LAMs does not define a homogeneous macrophage population, some of them exhibiting pro-inflammatory functions, although the majority corresponds to anti-inflammatory macrophages (77, 78). M2d macrophages are induced by stimulation of naive macrophages with IL-6 or by co-stimulation with an agonist of TLRs and of adenosine receptors (79, 80). M2d macrophages secrete significant amounts of anti-inflammatory cytokines and chemokines (IL-10, CCL18, CCL22…) that participate in shaping an anti-inflammatory environment. M2d macrophages bear the strongest immunosuppressive ability and promote angiogenesis and tumor progression. Indeed, activation of the adenosine receptor impairs the expression of pro-inflammatory and strongly increases the expression of anti-inflammatory cytokines such as CCL18 and CCL22 that induce the recruitment of naive T cells and Treg and the activation of T cells into Treg (81). M2d macrophages also express the ILT3 receptor on their surface, that can dimerize with a stimulatory receptor such as MHC-II or CD16 and thus acts as a natural inhibitor of these receptors (82). Ultimately, ILT3 expression strongly reduces the ability of macrophage to adopt a pro-inflammatory profile despite the presence of pro-inflammatory signals. In addition, M2d macrophages express VEGF, conferring pro-angiogenic properties that promote cancer cell growth (83).

### 3.3 The plasticity of macrophages

Once polarized, macrophages can still undergo profound adaptation of their phenotype. Thus, polarized macrophages can be reprogrammed *ex vivo* in response to different stimuli. For instance, repolarization of anti-inflammatory macrophages by LPS+INFγ increases the expression of pro-inflammatory markers and cytokines, while decreasing the expression of their anti-inflammatory counterparts. Conversely, repolarization of pro-inflammatory macrophages using IL-4 increases the expression of anti-inflammatory markers and cytokines (84, 85). *In vivo*, most studies have focused on the repolarization of TAMs into pro-inflammatory macrophages. For example, in a mouse model of breast cancer, metformin triggers repolarization of TAMs into pro-inflammatory macrophages (86). Therefore, the plasticity of polarized macrophages has paved the way for therapeutic intervention aiming at modifying their phenotype and function in different pathological settings. While a given subtype of anti-inflammatory macrophages can be repolarized into another type of macrophage, M2b macrophages cannot be repolarized into M1 macrophages under any conditions. The molecular mechanism blocking this repolarization is, at present, unknown (66).

### 3.4 Role of macrophages in cancer

**Figure 6:**
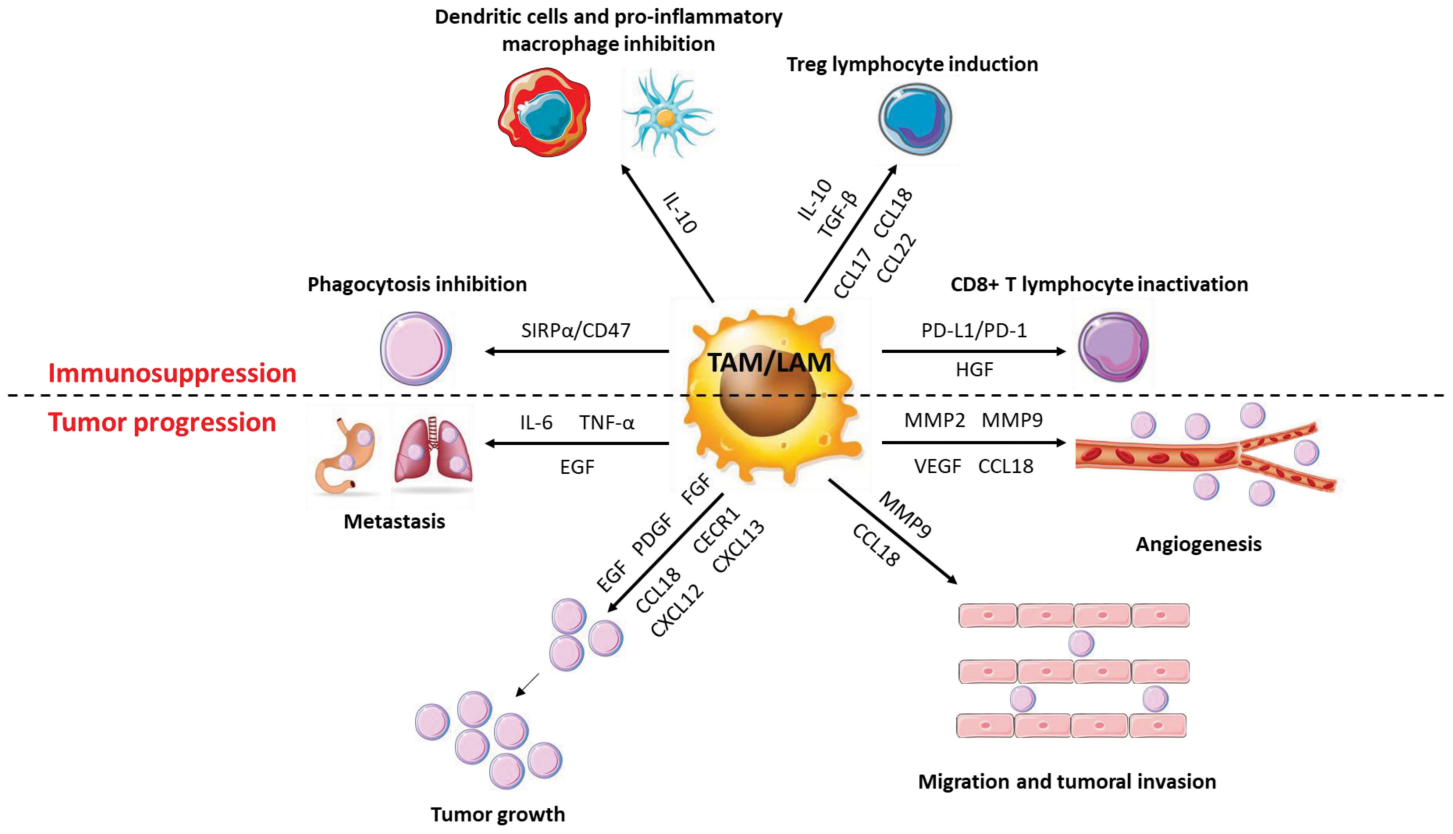
Role of macrophages in cancer progression. TAMs have a very diverse activity profile to promote cancer cells. They promote the growth and metastatic potential of cancer cells by secreting numerous molecules (cytokines, chemokines, growth factors, etc.) that act directly or indirectly on them. Thus, TAMs disrupt the anti-tumor response and promote angiogenesis, tumor growth and metastatic potential.

In many, if not all, solid cancers and in hematological malignancies, pro-tumor macrophages (TAMs and LAMs respectively) play a deleterious role for the organism and contribute strongly to tumor growth via different processes (**Figure 6**). In contrast, macrophages with a rather pro-inflammatory profile represent major players in the elimination of cancer cells. CD169^+^ macrophages (Siglec-1) can phagocytose dead cancer cells during the initial phases of tumor progression. They also present tumor antigens on their surface that activate CD8^+^ T cells involved in the mediation of an anti-tumor response by targeting and killing cancer cells. When the CD169^+^ macrophage population is depleted, CD8^+^ T cell activation is greatly reduced, as is the overall anti-tumor response. Thus, these CD169^+^ specific macrophages act as antigen-presenting cells and are of paramount importance to the CD8^+^ T cell anti-tumor response (87). More generally, pro-inflammatory macrophages, by producing nitric oxide or TRAIL, a ligand for DR4 and DR5 may induce tumor cell death. This anti-tumor effect mainly occurs during the early stages of tumor development, as pro-inflammatory macrophages are rapidly reprogrammed by cancer cells to promote their growth and expansion (88). For a long time, it was accepted that pro-tumor macrophages were exclusively derived from monocytes following their recruitment by cancer cells. However, over the last decade, a growing number of evidence have pointed to the role played by resident macrophages in tumor growth. Thus, in cancers, TAMs and LAMs constitute a highly heterogeneous population, with different functions and origins (89). Using mouse models, it has been established that the proportion of resident macrophages in TAMs and LAMs can be different depending on the cancer location. In a mouse model of glioma, microglia which are the resident macrophages in the brain was the main source of TAMs (90). In humans, the proportion of microglia composing TAMs population differs according to the type of glioma and seems less deleterious than monocyte-derived TAMs which display a greater immunosuppressive signature and are associated with higher mortality (91). The same picture could be drawn in pancreatic cancer, where most TAMs derived from resident macrophages of embryonic origin. These TAMs can proliferate, thus ensuring a constant increase in their number as well as their renewal. Their profile is also different from that of monocyte-derived TAMs, with a strong capacity to remodel the extracellular matrix, suggesting a role for these embryonic-derived TAMs in the metastatic capabilities of cancer cells. Furthermore, in a CCR2-deficient mouse model, inhibition of monocyte-derived macrophage recruitment is not sufficient to inhibit tumor progression while deletion of resident macrophages significantly reduces tumor progression, demonstrating the impact of resident macrophages in pancreatic cancer progression (92). In a mouse model of lung cancer, it was reported that monocyte-derived TAMs as well as interstitial macrophages of embryonic origin were present in the tumor while alveolar macrophages (the resident macrophages of the lung), were absent, suggesting either their progressive elimination during tumor development or a drastic change in their phenotype. It has been suggested that the various macrophage subpopulations found in the tumor displayed different functions, with monocyte-derived TAMs impacting cancer cell dissemination while TAMs of embryonic origin being involved in tumor growth (93). In a mouse model of breast cancer, characterization of TAM populations as well as their spatial localization demonstrated that TAM niches present within the tumor are modified throughout tumor development, exhibiting different macrophage populations depending on the stage of tumor evolution. For example, ductal macrophages, present in the ductal epithelium, accumulate in the tumor at the expense of stromal macrophages, which are confined to the healthy tissues surrounding the tumor. This contributes to the establishment of different macrophage niches within the breast tumor and surrounding tissue that support the generation of specific subpopulations of macrophages, with different functions and polarization status. In a model where the macrophage niches show little diversity, the profile of macrophages tends to be the same, indicating the importance of niches in the establishment of the heterogeneity of macrophages. Finally, it has been shown that in humans, TAMs also exhibit some heterogeneity suggesting the establishment of different macrophage niches during human breast cancer development (94).

Anti-inflammatory macrophages are likely the most represented immune cells in the extracellular environment of solid tumors. They are recruited by cancer cells and the environment of the tumor shapes their functions. Indeed, co-culture of macrophages with ovarian tumor cells leads to their polarization into TAMs and is accompanied by overexpression of anti-inflammatory cytokines and chemokines and angiogenesis-promoting growth factors like VEGF (95). In the TME, exosomes are produced by a wide variety of cell types including tumor cells, whose components cause the differentiation and polarization of monocytes into TAMs (96). These exosomes also block the differentiation of monocytes into dendritic cells to ensure a significant presence of anti-inflammatory macrophages and to limit immune response. These TAMs are strongly immunosuppressive and secrete high amounts of IL-10 and TGFβ and fail to express MHC-II, thus preventing the activation of T lymphocytes. In addition, they dampen T cell proliferation in a TGF-β-dependent manner (97). CSF-1 is a poor prognostic marker in multiple cancers, including pancreatic cancer and breast cancer. Indeed, CSF-1 primes the differentiation of monocytes into macrophages with an anti-inflammatory profile and favors the anti-inflammatory polarization of macrophages (98–100). MCP-1 which is involved in monocyte recruitment to the tumor is secreted by cancer cells and leads to the massive recruitment of monocytes that, once in the tumor microenvironment can differentiate and polarize into TAMs. Once polarized, TAMs are also able to secrete MCP-1 to amplify the recruitment of new monocytes, thus forming a permanent recruitment loop (101, 102). Finally, VEGF or Placental Growth Factor (PlGF) are also able to induce the recruitment and survival of monocytes in the tumor micro-environment (103, 104).

### 3.5 Functions of TAMs

TAMs exert numerous pro-tumoral effects among which immunosuppression, tumor growth, angiogenesis, and improvement of the metastatic potential of cancerous cells.

#### 3.5.1 Immunosuppressive functions

TAMs secrete huge amounts of IL-10 and TGFβ that promote the activation of T cells into Th2 or Treg at the expense of pro-inflammatory Th1 T cells (105). Within the tumor, Tregs inhibit other types of T cells and in particular cytotoxic T cells whose main function is to eliminate cancer cells. TAMs also secrete various chemokines allowing the recruitment of immune cells with immunosuppressive functions. Thus, CCL17 and CCL22 induce the recruitment of Th2 and Treg lymphocytes while CCL18 promotes the recruitment of naive T lymphocytes. Naïve T lymphocytes, while being able to be activated as Th2 or Treg, promote cell anergy when they are in a particularly anti-inflammatory and immunosuppressive environment as is the case within a tumor (106). In ovarian cancers, Tregs are recruited by TAMs via CCL22 and presence of Tregs is a marker of poor prognosis (107). The secretion of IL-10 by TAMs inhibits the expression of pro-inflammatory cytokines such as IL-12 or TNFα by macrophages and dendritic cells and reduces IFNγ expression by NK cells, thus contributing to make the tumor microenvironment anti-inflammatory and to limit the pro-tumor response of the immune cells. IL-10 also reduces the expression of CD80 and CD86 co-stimulatory receptors on the surface of macrophages and dendritic cells, decreasing their ability to activate cytotoxic T cells (108). Finally, TAMs express co-inhibitory molecules such as PD-L1 (b7-h1) or b7-h4 on their surface, which function to block T cell activation. PD-L1 is known to bind to the PD-1 receptor of active T cells, resulting in the abolition of their activity by suppressing the pro-inflammatory action of T cells. The co-inhibitory molecule b7-h4 has a similar mechanism of action to PD-L1 but, unlike PD-L1, the receptor for b7-h4 has not yet been identified. However, it has been determined that b7-h4 can block cytotoxic T-cell activity, like PD-L1. The expression of b7-h4 by TAMs is increased by IL-10 secreted by Treg lymphocytes (109–112).

#### 3.5.2 Function as promoter of tumor growth

TAMs express and secrete numerous factors that promote tumor growth, such as EGF (Epithelial Growth Factor), PDGF (Platelet-Derived Growth Factor), FGF (Fibroblast Growth Factor) or HGF (Hepatocyte Growth Factor) (113). In ovarian cancers, expression by TAMs of the EGF-R triggers cancer cell proliferation and promotes their migration (114). In phyllodes tumors, a rare form of breast cancer, a positive feedback loop is established between TAMs and myofibroblasts and promotes tumor progression. Myofibroblasts secrete the chemokine CCL5 which binds to the CCR5 receptor expressed on the surface of macrophages, stimulates the AKT pathway and induces the polarization of macrophages into TAMs. TAMs then secrete the chemokine CCL18, which promotes phyllodes tumor proliferation by inducing the differentiation of fibroblasts into myofibroblasts, which in turn secrete CCL5 (115).

#### 3.5.3 Functions in neo-angiogenesis

One of the main characteristics of solid tumors is the generation of new blood vessels to sustain their development according to increased supply of nutrients. TAMs promote angiogenesis by expressing numerous pro-angiogenic factors such as VEGF, BFGF (Basic Fibroblast Growth Factor), TNFα or metalloproteinases such as MMP2 and MMP9. The expression of these different molecules leads to the proliferation of endothelial cells, the remodeling of the extracellular matrix and the neo-vascularization of tissues in cancers (116, 117). Moreover, VEGF production is greatly increased when TAMs are stimulated with CSF-1 or CCL2, inducing increased angiogenesis when these two cytokines/chemokines are present in large amounts within the tumor (118). CCL18 secretion by TAMs, in association with VEGF, also promotes angiogenesis. Nevertheless, the mode of action of the chemokine CCL18 on VEGF production, and more generally on the induction of angiogenesis, has not yet been determined (119).

#### 3.5.4 Functions in promoting metastatic potential

The dissemination of cancer cells in the body (metastasis) is a marker of very poor prognosis in all types of cancers. An epithelial-mesenchymal transition (EMT) stage is necessary for cancer cells to spread in the body. During this step, cancer cells lose their cell-cell adhesion, acquire migratory properties, invade the extracellular matrix before transiting through blood vessels to form metastases (120, 121). TAMs are important players in the dissemination of cancer cells as they contribute to the degradation of the extracellular matrix by secreting proteases into the extracellular environment, including cathepsins or metalloproteinases (122). For example, the production of MMP9 by TAMs contributes to the metastatic potential of melanoma by restructuring the extracellular matrix (123). In lung cancers, TAMs secrete IL-6 and TNFα in response to TLR2 activation by versicane, a proteoglycan produced by cancer cells. Both cytokines are required for cancer cell dissemination by inducing low-grade inflammation that promotes extracellular matrix destruction (124). Excessive production of CCL18 by TAMs is correlated with increased aggressiveness of cancer cells. Indeed, CCL18 can bind to the PITPNM3 receptor of cancer cells to induce integrin clustering that enhances the invasion of the extracellular matrix by cancer cells leading to their migration and metastasis (125). Finally, TAMs can directly promote EMT by secreting EGF. EGF binds to the EGFR, resulting in activation of the ERK1/2 pathway and expression of various markers of EMT including vimentin and Slug (126).

### 3.6 LAMs in leukemia

The presence of macrophages within hematopoietic stem cell niches in the BM has only been recently documented (127–129). In this line, the presence of leukemia-associated macrophages (LAMs) within the BM was demonstrated in a mouse model of T-ALL (T Acute Lymphoblastic Leukemia). In this model, LAMs display unique characteristics that differ significantly from TAMs, including both a pro- and anti-inflammatory gene expression profile. LAMs produce lower amounts of CSF-1, TGFβ, and VEGF than TAMs but exhibit higher amounts of IL-1β, IL-6, and CXCL11 (76). Despite these differences, LAMs share the same functions and mechanism of action as TAMs, i.e., pro-tumor effects such as immunosuppression or cancer cell proliferation. Nevertheless, the role of LAMs in the metastatic potential of hematologic cancer cells is still poorly established (80). In Acute Myeloid Leukemia (AML), cancer cells induce the differentiation and polarization of monocytes into LAMs via Gfi1, a transcription factor involved in macrophage development (130). Moreover, it was established that LAMs are associated with an unfavorable prognosis in AML patients (131). In T-ALL and chronic lymphocytic leukemia (CLL), the generation of LAMs is dependent on M-CSF. Indeed, inhibition of CSF1R leads to a defect in anti-inflammatory macrophage infiltration in BM as well as reduced leukemia progression (132–135). In CLL, LAMs, also known as CLL-nurse like cells (NLCs), promote the survival and proliferation of cancer cells via the expression of the chemokines CXCL12 and CXCL13 and impairs treatment-mediated apoptosis (136).

## 4 Targeting macrophages in cancer

### 4.1 In solid tumors

**Figure 7:**
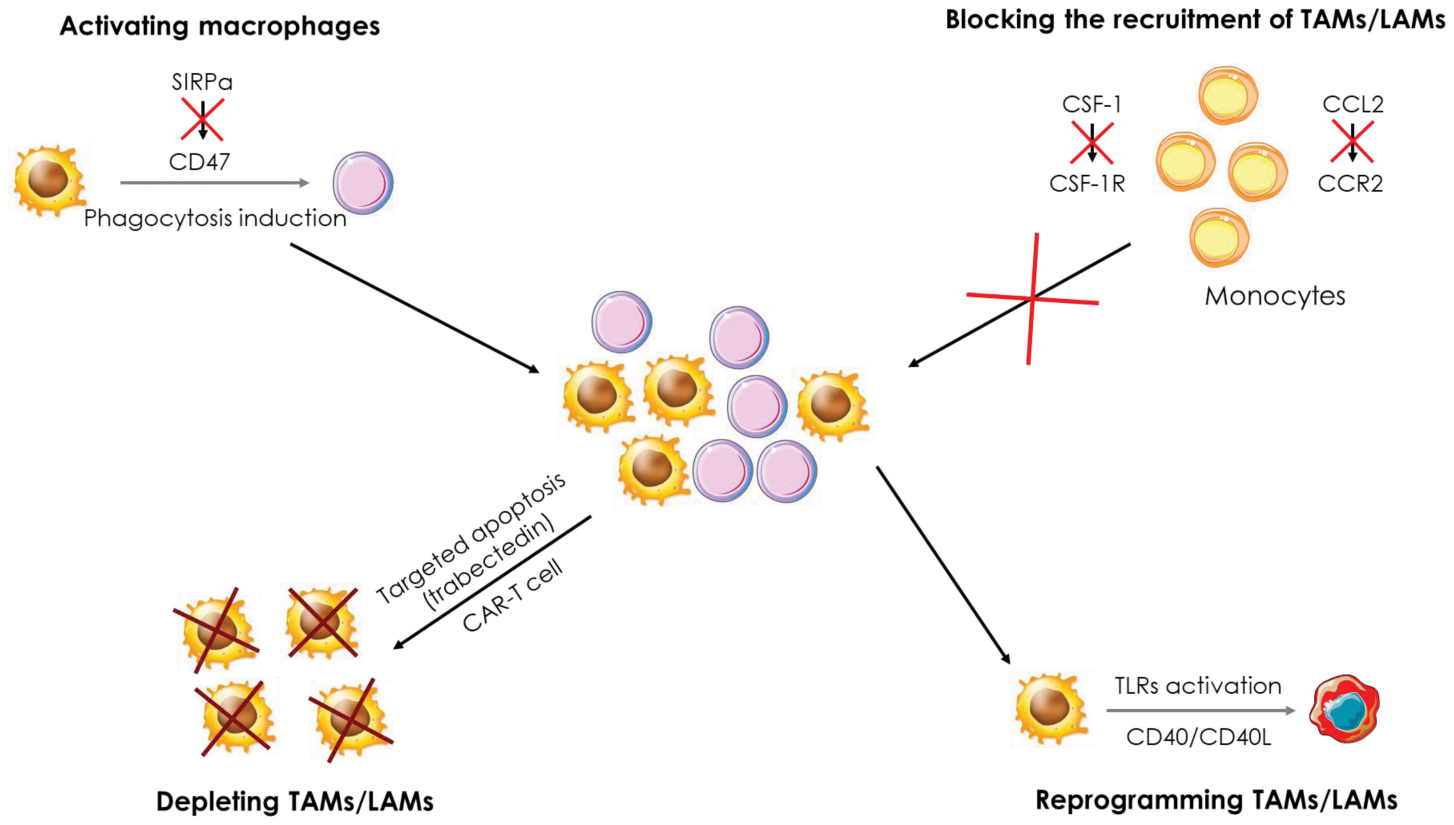
Therapeutic strategies targeting macrophages in cancer. Several therapeutic strategies targeting TAMs/LAMs are being developed to treat cancers. i) Blockade of monocyte recruitment by inhibiting the CCL2/CCR2 and CSF-1/CSFR1 axes, ii) Depletion of TAMs/LAMs using apoptosis inducers and CAR-T cells, iii) Activation of macrophages through the inhibition of the SIRPα/CD47 axis to induce phagocytosis of cancer cells and iv) reprogramming of TAMs/LAMs by activation of the CD40/CD40L axis and TLRs.

The identification of the deleterious role of TAMs in cancer has paved the way for the development of therapeutic strategies to modulate their functions. These strategies aim at limiting their recruitment or/and depleting, modulating, or reprogramming them within the tumor (**Figure 7**). In this section, we will essentially present the different results obtained in recent years in pre-clinical models and in clinical studies.

#### 4.1.1 Recruitment, generation, and depletion of TAMs

The CCL2-CCR2 axis is the main pathway for the recruitment of TAMs within the tumor. Targeting this pathway allows to reduce the number of TAMs recruited and to promote a pro-inflammatory response and a reduction of tumor growth. CCL2 deletion in many types of cancers (lung, endometrial, breast, etc.) leads to a drastic decrease in the number of TAMs infiltrating the tumors because of defects in monocyte recruitment (137–139). The use of CCL2 neutralizing antibodies in a mouse model of xenografted human renal cancer strongly reduces the presence of TAMs, xenograft growth and blood vessel density (140). In parallel to the pre-clinical trials, clinical studies were conducted targeting either CCL2 or CCR2 and CCR5 receptors. For example, a phase II study in prostate cancer patients was managed to investigate the efficacy of Carlumab, an antibody directed against CCL2. Although Carlumab was well tolerated by patients, it only resulted in transient suppression of chemokine effects, did not block the CCL2/CCR2 pathway, and exhibit no anti-tumor effect (141). Two phase 1b/2 studies in patients with pancreatic cancer were launched to test the tolerability and efficacy of a CCR2 antagonist, PF-04136309. The first one showed that PF-04136309, in combination with Folforinox chemotherapy, was well tolerated by patients and induced a decrease in the number of TAMs in tumors (142). The second study established first that PF-04136309 in combination with nab-paclitaxel and gemcitabine exhibits some lung toxicity in patients. In addition, the decrease in the number of TAMs in tumors was observed in only 2 of the 21 patients enrolled in the study (143), two observations that preclude further utilization of this combo in the clinic. By contrast, the PF-04136309/Folforinox combination which shows acceptable results in phase 1b/2 would warrant further investigation. Finally, a recent phase I study in patients with colorectal cancer showed no benefit of the anti-PD-1 drug Pembrolizumab and the CCR5 antagonist Maraviroc combination (144). Nevertheless, additional phase I and II studies involving blockade of the CCL2/CCR2 pathway are underway in various types of cancers (145).

CSF-1 is another cytokine that allows the recruitment of TAMs to the tumor and several therapeutic strategies aiming at blocking the activation of the CSF-1/CSF1R pathway are currently investigated. For instance, CSF1R inhibition was shown to exhibit anti-tumor activity in AML by blocking paracrine signals from support cells (146) and disruption of CSF-1 receptor signaling by BLZ945 inhibits growth of AML cells with inversion of the chromosome 16 (147). Emactuzumab is a human monoclonal antibody to CSF1R that blocks its dimerization and activation that was found to trigger the specific depletion of anti-inflammatory CSF1R+ macrophages *in vitro* and *in vivo* in the primate Macaca Fascicularis. However, macrophages generated following stimulation with CSF-2, which exhibit a global pro-inflammatory profile, are not affected by Emactuzumab. In mice, a murine antibody against CSF1R, named 2G2, was generated to mimic the action of Emactuzumab. In colorectal cancer and fibrosarcoma models, 2G2 induced a drastic reduction in the number of TAMs in the tumors associated to a greater infiltration of neutrophils and cytotoxic T lymphocytes, impeding tumor development. Finally, a phase I study was conducted in patients with pigmented villonodular synovitis (SVN or diffuse-type giant cell tumor (Dt-GCT)), a rare type of tumor. The administration of Emactuzumab improves the clinical status of patients inducing partial responses. This study was completed by administering Emactuzumab to patients with various other types of solid tumors (lung cancer, liver cancer, …) with similar results. Thus, targeting the CSF-1/CSF1R pathway appears to represent an attractive therapeutic strategy in the treatment of cancers (148, 149). A phase I study in glioblastoma patients evaluated the toxicity and efficacy of BLZ945, in combination with PDR001, an anti-PD1 antibody. A good tolerance of the combination was established combined with encouraging anti-tumor activity, which led to the initiation of a phase II study that is currently underway (150). In a mouse model of lung cancer, BLZ945 fails to induce depletion of CSF1R+ TAMs but causes remodeling of the tumor microenvironment in which immune cells present within the tumor, such as NK or T cells, secrete large amounts of IFN-γ. In addition, dendritic cells were found to express high level of IL-12, together promoting an effective anti-tumor response (151). PLX3397 is a CSF1R inhibitor that induces TAMs depletion in a mouse model of osteosarcoma. This depletion results in a slowdown of tumor growth and a decrease in the number of metastases, while reducing the number of Treg and improving tumor infiltration by cytotoxic T lymphocytes (152). A phase I study in patients with sarcoma established that PLX3397 in combination with Sirolimus an mTOR pathway inhibitor, was well-tolerated. In these patients, the number of TAMs was decreased in the tumors, supporting an advancement of this combination into phase II (153). In a phase II study, the combination of AMG820, an antibody to CSF1R and Pembrolizumab showed no significant effect since no treated patients exceeded the predefined efficacy threshold and only one-third of patients experienced stable disease progression or partial response (154). Other phase I and II studies involving blockade of the CSF-1/CSF1R pathway are underway in different types of cancers (145). Trabectedin, a pro-apoptotic molecule, is currently used in many clinical trials because it specifically induces apoptotic death in macrophages, including TAMs. This apoptotic effect is due to the activation of the TRAIL signaling pathway which leads to the activation of caspase-8 and ultimately to macrophage apoptosis. This apoptosis is necessary for the anti-tumor activity of trabectedin (155). Recently, a CAR-T cell (Chimeric Antigen Receptor T cell) possessing a CAR recognizing the folate receptor in mouse models of ovarian, colon or melanoma cancers showed promising results. Indeed, the folate receptor is mainly expressed by anti-inflammatory TAMs and its targeting via CAR-T cells allows to specifically suppress this macrophage population and increase the infiltration of immune cells such as cytotoxic T lymphocytes and pro-inflammatory monocytes. In addition, tumor progression was significantly slowed down, and survival of mice was improved. In conclusion, this new type of treatment could be very effective in specifically targeting TAMs while preserving other macrophage populations (156).

#### 4.1.2 Activation/inactivation and reprogramming of TAMs

The CD40/CD40L axis is a pathway involved in the activation of pro-inflammatory macrophages. When CD40L, expressed on the surface of activated T cells, binds to the CD40 receptor, it triggers the production of various factors such as TNFα or ROS. Thus, the CD40/CD40L pathway participates in the anti-microbial and anti-tumor activity of macrophages. In addition, it also stimulates T-cell induced immunity, thus initiating a positive feedback loop. Different strategies have been developed to trigger the activation of this pathway and to force macrophages to exert anti-tumor activity. A first phase I study with a CD40 agonist monoclonal antibody (CP-870,893) in patients with pancreatic cancer in combination with gemcitabine gave appreciable results. Indeed, the combination was well tolerated by the patients and showed promising anti-tumor effects, justifying its progression to phase II (157). In a mouse model of pancreatic cancer, the use of a CD40 agonist, FGK.45, induces T-cell infiltration into the tumor. In combination with an anti-PD1, the tumor even shows regression, dependent on the infiltration of CD8^+^ and CD4^+^ T cells. CD40 activation leads to an increase in CCL5 expression by TAMs allowing the recruitment of CD4^+^ T cells into the tumor, which are essential for tumor regression. In addition, macrophages transiently express CD86 and MHC-II after treatment with a CD40 agonist, promoting pro-inflammatory T cell activation (158). The combination of 2G2 and FGK.45 not only efficiently depletes TAMs, but also induces their pro-inflammatory activation prior to their death. These results, obtained in a mouse model of colon cancer, highlighted the transient hyperactivation of TAMs that leads to the recruitment and activation of cytotoxic T cells and ultimately to tumor regression (159). In addition, a phase I clinical trial was conducted combining Emactuzumab with the CD40 agonist antibody Selicrelumab. This treatment administered to patients with various types of solid tumors showed an acceptable safety profile and an increase in the number of cytotoxic T lymphocytes in the tumors. However, no strong clinical response was achieved, 40% of patients showing stabilization of the pathology but no patient exhibiting improvement (160). In patients with metastatic pancreatic cancer, the combination of APX005M, a CD40 agonist antibody, with the reference treatment gemcitabine + nab-paclitaxel, with or without immunotherapy (using nivolumab, an anti-PD1), was well tolerated by patients. This treatment induced a clinical response in more than half of the patients (14 of 24). A phase II study is underway and if the results observed in phase I are confirmed in phase II and III assays, this combination could replace the current chemotherapy used for patients with metastatic pancreatic cancer (161). In addition, numerous phase I studies are underway in a variety of solid tumor types to determine the effects of CD40 agonists on tumor growth progression (145).

Another way to activate macrophages is to target TLRs, which, upon activation, promote the pro-inflammatory activity of macrophages and suppress their immunosuppressive activities. The gold standard treatment for bladder cancer patients is BCG (bacillus Calmette-Guérin) which stimulates TLR2 and TLR4 to induce a pro-inflammatory response in macrophages. Nevertheless, relapses are frequent, and some patients do not respond to BCG treatment. Therefore, there is a need to improve this treatment to trigger a better response in patients (162). Recently, a phase I study tested the efficacy of a percutaneous BCG primo-injection 21 days prior to intravesicular injection, with the goal of “priming” TAMs. The primary injection was well tolerated but some patients failed to respond to treatment, suggesting a mechanism of resistance to BCG (163). In a mouse model of breast cancer, the combination of IFN-γ and MPLA (Monophosphoryl Lipid A), a TLR4 ligand, results in the reduction of tumor growth and metastatic potential of cancer cells via reprogramming of TAMs into pro-inflammatory macrophages expressing TNFα and IL-12. The secretion of these two factors by the reprogrammed macrophages activates cytotoxic T lymphocytes that participate in the elimination of cancer cells. Furthermore, in a mouse model of ovarian cancer, the combination of IFN-γ and MPLA increases the sensitivity of cancer cells to chemotherapies. Currently, IFN-γ and MPLA are used separately in clinical studies and their combination could lead to a new therapeutic approach to induce reprogramming of TAMs in cancers (164). Another pre-clinical study shows an anti-tumor effect after treatment of mice with lung cancer or fibrosarcoma with the combination of a TLR3 agonist poly(I:C) and TLR8 agonists, R837 or R848. This study was motivated by the fact that these agonists are used in clinical studies individually. The combination of a TLR3 and TLR8 agonist repolarizes TAMs into pro-inflammatory macrophages, reduces tumor growth and increases T cell infiltration into tumors. Thus, combining these two agonists represents a new therapeutic opportunity and a phase I study would be interesting to conduct (165). A phase I study in patients with various types of cancers including melanoma and kidney cancer refractory to anti-PD1 showed that a nanoplexed form of poly(I:C), BO-112, in combination with an anti-PD resulted in an anti-tumor response with partial responses in 10% of patients and stabilization of the pathology in more than one third of patients. BO-112, in combination with an anti-PD1, could therefore represent a new therapeutic strategy to combat anti-PD1 resistance (165). Similarly, the use of a TLR9 agonist, CMP-001, in combination with an anti-PD1 in patients with metastatic melanoma overcomes resistance to anti-PD1. The TAMs are reprogrammed and secrete IFN-γ which allows the recruitment of T lymphocytes and a reduction of the tumor in 25% of the patients in this study. Considering these promising results, a phase II study is underway (166). Numerous other phase I to III studies involving TLR agonists are underway as monotherapy or in combination with other molecules. Overall, these treatments are well tolerated by patients and show interesting clinical effects, in particular stabilization of diverse pathologies. Currently, none of these treatments has been approved to treat cancer, but it is not excluded that some of them will complete the arsenal of cancer treatment in the coming years (145, 167–169).

Specific markers can be expressed on the cell surface that are referred to as “don’t eat me” signals. One of these signals is CD47, which is overexpressed in many cancers. Expression of CD47 prevents cancer cells from being phagocytosed and thus promotes tumor growth. CD47 is recognized by the SIRPα protein expressed on the surface of monocytes/macrophages and neutrophils and multiple pre-clinical and clinical trials are underway to overcome the inhibition of phagocytosis induced by the CD47/SIRPα axis. The use of an anti-CD47 antibody not only enhances phagocytosis of cancer cells by macrophages but also facilitates antigen presentation by macrophages to ultimately activate cytotoxic T cells (170). *In vitro*, the use of an anti-CD47 antibody, Hu5F9-G4, increases the phagocytic capacities of pro-inflammatory as well as anti-inflammatory macrophages. *In vivo*, CD47 blockade results in significant pro-inflammatory polarization of macrophages in mice xenografted with human glioblastoma and enhanced phagocytosis of tumor cells. A clinical study is underway regarding the use of Hu5F9-G4 in colorectal cancer patients (171). Two other preclinical studies in mouse models of lung cancer and breast cancer show similar results. In the lung cancer model, Hu5F9-G4 inhibited tumor growth by promoting the phagocytic abilities of macrophages (172). In the breast cancer model, Hu5F9-G4 was used in combination with trastuzumab, an anti-HER2 antibody. Indeed, nearly 30% of breast cancer patients have HER2 overexpression, and trastuzumab is one of the gold standard treatments for HER2-overexpressing breast cancers. However, relapses are frequent and new therapeutic strategies must be developed to fight these relapses more effectively. The combination of Hu5F9-G4 and trastuzumab was shown to reduce tumor growth and overcome the resistance of cancer cells to trastuzumab. This combination holds great promise, particularly for patients with trastuzumab resistance (173). Currently, results of phase I and II studies investigating the efficacy of anti-CD47 in different types of cancer show promising results, particularly in combination with reference treatments where they lead to a decrease in toxicity (145).

#### 4.1.3 Chimeric antigen receptor macrophages

CAR-M represents a new weapon in the armamentarium of therapeutic strategies available to fight solid and hematopoietic cancers. Based on the principle of CAR-T cells, it is assumed that CAR-M cells would be more effective than CAR-T cells in patients with solid tumors. Indeed, CAR-T cells are very effective in hematological cancers but show limited results in solid tumors. In a mouse model of breast cancer, the administration of CAR-M cells expressing an anti-HER2 antibody and the transmembrane and intracellular domain of CD147 allows to specifically target cancer cells expressing HER2. The presence of CAR-M cells inhibits tumor growth and is associated with a massive infiltration of T cells in the tumor. Activation of CD147 also promotes the production of MMPs that degrade the extracellular matrix and participate in the destabilization of the tumor microenvironment (174). Injection of another CAR-M targeting HER2 into mice carrying lung metastases significantly reduced tumor burden and increased mouse survival. In addition, CAR-M can remodel the tumor microenvironment to create a pro-inflammatory climate that promotes T cell recruitment and activity. *In vitro*, CAR-M modify the phenotype of anti-inflammatory macrophages by reprogramming them into pro-inflammatory macrophages. This observation suggests that the same phenomenon takes place *in vivo*, with TAMs reprogrammed into pro-inflammatory macrophages (175). A clinical study is underway to test the efficacy of this CAR-M in patients. This new strategy seems promising and, although the number of pre-clinical and clinical studies is still very low, it would be of cardinal interest if CAR-M cells prove to be as effective in solid tumors as CAR-T cells in hematopoietic malignancies (176).

### 4.2 In leukemia

Many of the treatments targeting LAMs in leukemia involve the use of an anti-CD47 antibody. Bispecific antibodies have been developed to be more effective against cancer cells. RTX-CD47 is a bispecific antibody targeting CD20, a molecule overexpressed on the surface of leukemic B lymphocytes, and CD47. *In vitro*, RTX-CD47 induces phagocytosis by macrophages of B lymphocytes from different malignant B lymphocyte cell lines in a co-culture system. Moreover, the use of this bi-specific antibody on healthy donor cells does induce their phagocytosis by macrophages, demonstrating a strict specificity of this antibody for CD20 overexpressing cells. In combination with three anti-cancer therapies, daratumumab (anti-CD38), alemtuzumab (anti-CD54), and obinutuzumab (anti-CD20), TRX-CD47 synergistically increases the phagocytic potential of macrophages and allows for more efficient elimination of malignant B cells (177). CD33 is expressed on the surface of all myeloid cells but its expression significantly increased in leukemic blasts. This is why MBD004, a CD33/CD47 bi-specific antibody was used in a mouse model of AML. This bispecific antibody was shown to induce a reduction in the number of blasts. HMBDOO4 in addition to acting at the level of blasts, also reduced the hemagglutination of erythrocytes observed when using an anti-CD47 alone and therefore increases tolerability in patients. HMBD004 facilitates the specific phagocytosis of blasts by macrophages, thus leading to a decrease in tumor mass. A phase I clinical trial is underway to determine the toxicity of HMBD004 in patients with AML (178). The development of a bi-specific B-cell-targeting antibody, NI-1701, was conducted to allow targeting of CD20-resistant malignant B cells in B-CLL (B-Chronic Lymphocytic Leukemia) and B-ALL (B-Acute Lymphoblastic Leukemia). NI-1701 combines an anti-CD47 and an anti-CD19 antibody (specific marker for B lymphocytes). The use of NI-1701 in several mouse models of B-cell lymphoma and leukemia significantly reduced the growth of blasts by facilitating their phagocytosis by macrophages. Furthermore, in a primate model, NI-1701 was found to be well tolerated and could therefore entered clinical trials (179). In a phase I study, the lack of efficacy of CC-90002, an anti-CD47 antibody led to the discontinuation of the clinical trial that targeted patients with refractory or relapsed AML or high-risk myelodysplastic syndromes. Indeed, although treatment was well-tolerated, CC-90002 failed to induce a strong and durable response (180). Nevertheless, the use of anti-CD47 antibodies in combination with standard of care for different diseases has shown globally promising results. A phase I-b study combining Magrolimab, an anti-CD47, with azacitidine in patients with myelodysplastic syndromes or AML shows very promising results with a percentage objective response rate of 100% in MDS and 69% in AML. The treatment was also well tolerated by patients and was characterized by the complete loss of blast cells in the BM, particularly in TP53-mutated patients (181, 182). A phase III study is currently enrolling patients to investigate the efficacy of Magrolimab + azacitidine treatment in patients with high-risk MDS (183). A phase Ib study in patients with mutated TP53 MDS treated with Magrolimab + Azacitidine has also been initiated and preliminary results show a durable response and increased overall survival (184). A phase III study comparing this treatment to the current standard of care is also underway (181). Other phase I, II or III clinical trials have been initiated to test the efficacy and toxicity of different anti-CD47 antibodies as a single agent or in combination with different reference treatments (185). Finally, a phase I study in patients with pediatric leukemia showed that GNKG168, a TLR9 agonist, could be an interesting therapeutic approach. Indeed, the expression of genes involved in the regulation of blasts was decreased, suggesting a beneficial role of GNKG168 in tumor progression. However, further research needs to be conducted to determine whether TLR9 activation is beneficial for pediatric leukemia patients (186).

## 5 Future perspectives

The versatility of macrophages can be envisioned as a promising opportunity to design novel therapies. In this line, targeting and/or reprogramming immunosuppressive macrophages during cancer progression could be of utmost importance to improve current treatments and in particular immunotherapies. To achieve this goal, a better knowledge of the molecular mechanisms of macrophage generation, activation and polarization will undoubtedly help identifying signaling pathways and vulnerabilities for TAMs or LAMs targeting and elimination/reprogramming in solid and hematopoietic tumors. In this context, several strategies to prevent anti-inflammatory macrophage-mediated immunosuppression are currently being tested in different clinical trials. These clinical strategies aimed at limiting TAMs or LAMs recruitment and/or depleting, modulating, or reprogramming them within the tumor. Finally, the identification by our group of a protease cascade CTSB>Caspase-8>Caspase-3/7>Substrates required for the differentiation of monocytes and the persistence of this pathway during M2 macrophage polarization may open new avenues to manipulate macrophage plasticity to promote solid and hematopoietic tumor elimination.

## Author contributions

PC, EK, MB, CS, CF and GR were all involved in the concept, design, and critical review of the content of this article. PC makes the figures. AJ and PA write the paper. All authors agree to the accuracy and integrity of this work as presented. All authors contributed to the article and approved the submitted version.
